# Characterizing Wildland Firefighters’ Thermal Environment During Live-Fire Suppression

**DOI:** 10.3389/fphys.2019.00949

**Published:** 2019-08-02

**Authors:** Belén Carballo-Leyenda, José G. Villa, Jorge López-Satué, Jose A. Rodríguez-Marroyo

**Affiliations:** ^1^VALFIS Research Group, Institute of Biomedicine, University of León, León, Spain; ^2^Empresa de Transformación Agraria (TRAGSA), Madrid, Spain

**Keywords:** thermal exposure, heat flux, thermal dose, heat stress, skin burn, attenuation factor

## Abstract

Wildland firefighters work under adverse environments (e.g., heat and fire exposure), which contribute to increasing the heat strain. Despite this there is a paucity of knowledge about the thermal environment in real wildfire suppression scenarios. Therefore, the main purpose of this study was to characterize the environmental thermal exposure and the risk of heat burn injuries during real wildfire suppression (*n* = 23). To characterize the wildland firefighter’s (*n* = 5) local thermal exposure, measurements of air temperature and heat flux were performed. Heat flux measurements were made using four thin-planar heat flux sensors. Two were affixed on the outer surface of the garment on the left chest and thigh. Two other sensors were placed on the inner surface of the fabric in parallel to those placed externally. Four thermal classes were defined based on the heat flux across the inner sensors (≤1000, ≤5000, ≤7000, and >7000 W⋅m^–2^). The risk of pain and first-degree burns were calculated using the dose of thermal radiation method. The inner sensors mean and maximum heat flux and environment temperature were 286.7 ± 255.0 and 2370.4 ± 3004.5 W⋅m^–2^ and 32.6 ± 8.9 and 78.0 ± 8.9°C, respectively. Approximately 81, 15, and 3.5% of the exposure time the heat flux was ≤1000, >1000–5000, and >5000 W⋅m^–2^, respectively. The highest average and maximum thermal dose values were ∼94 and ∼110 (kW⋅m^–2^)^4/3^⋅s. In conclusion, the thermal exposure obtained may be considered light. However, high thermal exposure values may be obtained in punctual moments, which can elicit first-degree burns.

## Introduction

During wildfire suppression, wildland firefighters carry out demanding tasks under adverse environmental conditions (Rodríguez-Marroyo et al., 2012). Different factors such as the environmental work conditions (Budd et al., 1997; Cuddy et al., 2015), wearing the personal protective equipment (Carballo-Leyenda et al., 2017, 2018) and the type of work performed (Rodríguez-Marroyo et al., 2011, 2012) contribute to considerably increase the heat strain. In these circumstances, an excessive increase in the thermophysiological demands could lead to an impaired physical performance, heat exhaustion, or even heat stroke (Cuddy and Ruby, 2011; Carballo-Leyenda et al., 2018).

In recent years, the thermophysiological impact of personal protective equipment on wildland firefighters under laboratory conditions has been studied (Carballo-Leyenda et al., 2017, 2018). The physiological effort performed in real scenarios and the environmental temperature in which they have to perform their work has also been analyzed (Rodríguez-Marroyo et al., 2012; Cuddy et al., 2015). However, to our knowledge no previous studies have investigated the exposure to heat flux that wildland firefighters have to withstand during real wildfire situations. This variable can significantly raise the environmental thermal load during fire suppression (Bröde et al., 2010; Willi et al., 2016) In addition, a direct exposure to thermal radiation and convection emitted by the flames elevates the risk of heat injuries (Rossi, 2003; Raimundo and Figueiredo, 2009). It has been reported in American wildland firefighters that 66% of injuries during wildfire suppression (between 2003 and 2007) were heat burns (Britton et al., 2013). Under these circumstances the thermal protective clothing and equipment have a main role on the subjects’ safety. Although the protective clothing is manufactured using high thermal resistance materials (i.e., Nomex® and Kevlar®) and it is certified according to normative laboratory tests (International Standardization Organization [ISO] 15384, 2018), its protective performance may vary in real conditions (Song et al., 2011). Factors such as the magnitude and time of heat flux exposure, the fit of the garments (i.e., layer of air between the fabric and the skin) or the moisture content of the fabric can influence the amount of heat transmitted inside the clothes and the incidence of burns (Keiser and Rossi, 2008).

Only early studies have analyzed the heat flux during simulated wildfires (King, 1962; Budd et al., 1997). These works reported radiant heat fluxes between 0.4 and 8.6 kW⋅m^–2^ (King, 1962; Budd et al., 1997) and radiant temperatures of 33–96°C (Budd et al., 1997). Subsequent studies have focused on applying mathematical models to predict the heat flux and fire related injury for the establishment of safety zone sizes during wildfire suppression (Butler and Cohen, 1998; Zárate et al., 2008; Parsons et al., 2014). The knowledge of the thermal environment wildland firefighters are exposed to, may help to understand the thermal stress they have to experience and the risk of heat burn injuries (Rossi, 2003). Therefore, the main aim of this study was to characterize the environmental thermal exposure and the risk of heat burn injuries during real wildfire suppression. Secondarily, the effect of protective clothing in attenuating the fire heat flux was analyzed.

## Methods

### Participants

Five voluntary male wildland firefighters from different Spanish helitack crew bases took part in this study (age: 28 ± 1 years; body mass: 76.2 ± 0.9 kg, height: 175.5 ± 0.5 cm). Volunteers had a minimum experience of 2 years in wildfire suppression, and all of them were familiar with live-fire policies and procedures. Written informed consent was obtained from the subjects before starting the study. The experimental protocol was developed in accordance with the guidelines of the Helsinki Conference for research on human subjects and was approved by the Ethics Committee of the University of León, León, Spain.

### Experimental Design

**FIGURE 1:**
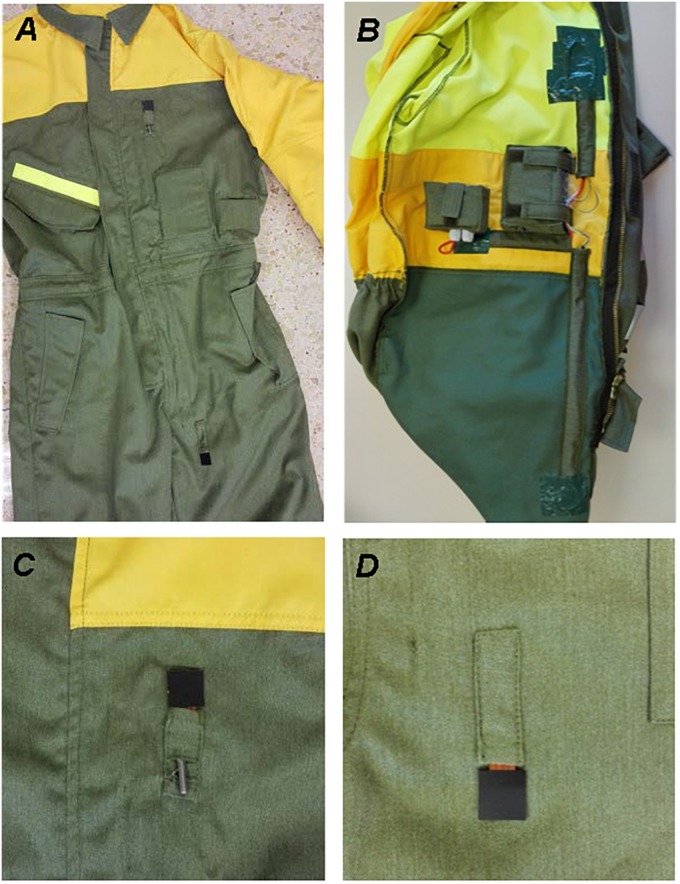
**(A)** Heat flux sensors and temperature probe on the outer surface of the protective suit. **(B)** Inside layout of sensors and dataloggers. **(C)** Detail of outer heat flux sensor and temperature probe in the chest. **(D)** Detail of the outer heat flux sensor in the thigh.

Thirty-eight wildfires were recorded during four summer seasons (i.e., June – October). To characterize the wildland firefighter’s local thermal exposure, individual measurements of air temperature and heat flux through each wildfire suppression event were performed. Ten protective suits were directly customized by the manufacturer (Confecciones Oroel, La Muela, Zaragoza, Spain), allowing the sensors and dataloggers to be safely and solidly placed using the customized holes, ducts and pockets (Figure 1).

When the fire alarm was received, the participants put on the personal protective equipment (i.e., helmet, gloves, mid-calf leather boots, and neck shroud) which included the protective suit (65% fire retardant viscose, 30% Nomex^®^, and 5% Kevlar®). The heat flux and the ambient temperature were continuously measured from the exit to the return to the base. The suppression time was calculated without accounting for the displacements to or from the wildfire.

### Measurements

Heat flux (the speed of thermal energy transfer) measurements were made using four thin-planar black coated heat flux sensors (Captec Enterprise, Lille, France; dimensions: 20 mm × 20 mm; thickness: 0.4 mm; heat flux range: ±50 kW⋅m^–2^; nominal sensitivity: 3.08–3.82 μV⋅W^–1^⋅m^–2^) which measured the combined radiative and convective heat flow. Following the manufacturer’s instructions, two sensors were affixed on the outer surface of the garment on the left chest and thigh, using its adhesive back surface (Figure 1). Two other sensors were placed on the inner surface of the fabric in parallel to those placed externally on the chest and thigh (Figure 1). These locations were chosen to characterize the heat flow that a wildland firefighter might face when suppressing flames with hand tools. The heat flux sensors were placed with the receiving surface facing outward in the direction of the greatest expected incident radiation (Raj, 2008). The positive heat flux values were considered as heat gain, while the negative values were considered as heat losses. The sensors were connected to a four-channel data logger (QuadVolt ± 100 mV, Madgetech, Warner, NH, United States; nominal range: ± 30 kW⋅m^–2^; resolution: 1.43 W⋅m^–2^). The heat flux was recorded continuously at a sampling rate of 5 s (0.2 Hz) from the exit to the return from the fire event.

Air temperature was measured with a resistive temperature probe Pt100 [ControlTemp, Santa Perpetua de Mogoda, Barcelona, Spain; nominal range: −200°C and 650°C; accuracy: ±(0.30+0.005 × t)°C], that was placed on the left side chest of the garment surface. The temperature probe was connected to a specific data logger (LogBox AA IP65; Novus, Porto Alegre, Brazil; nominal range: −40°C and 70°C; accuracy: 0.2% FS) that was placed in an inside pocket of the protective suit created for that purpose (Figure 1). Air temperature was continuously measured at a sampling rate of 5 s (0.2 Hz). The temperature data in the geographical area of the wildfire provided by the State Meteorological Agency were recorded and compared to the work environment temperature.

The attenuation factor (AF) of the protective clothing was calculated as the heat flux received in the outer sensors (q_out_, W⋅m^–2^) relative to the heat flux received in the inner sensors (q_in_, W⋅m^–2^) using equation 1 (Raj, 2008), while the percentage of attenuation was calculated using equation 2:

(1) $$\mathrm{AF}=\left(\frac{q_{\mathrm{out}}}{q_{\mathrm{in}}}\right)$$

(2) $$\mathrm{AF}\left(\%\right)=\left[\frac{q_{\mathrm{out}}}{q_{\mathrm{in}}}-1\right]\times 100$$

Following the methodology described to define structural firefighters’ thermal environment (Krasny et al., 1988; Foster and Roberts, 1994; Rossi, 2003), four thermal classes were defined based on the heat flux recorded in the inner sensors: Class 1, heat flux ≤1000 W⋅m^–2^; Class 2, >1000–≤5000 W⋅m^–2^; Class 3, >5000–≤7000 W⋅m^–2^; Class 4, >7000 W⋅m^–2^. A heat flux of 1000 W⋅m^–2^ corresponds to the heat flux received on a summer day and is assumed to be harmless for any exposure time (Raj, 2008). A heat flux of 5000 W⋅m^–2^ may cause pain after 15 s and second-degree burns at exposures of 30 s (Raj, 2008). There is a consensus among several international agencies to consider this threshold as the limit of exposure to thermal radiation for people without protection (Raj, 2008). When firefighters wear Nomex cloth (210 g⋅m^–2^), second degree burns might occur after 90 s at incident radiant heat fluxes of approximately 7000 W⋅m^–2^ (Butler and Cohen, 1998; Zárate et al., 2008).

The effective duration of heat exposure was calculated when positive heat flux was recorded. The weight of heat exposure was calculated as the ratio of exposure time to the total time of work in the wildfire suppression area. Displacements to or from the fire area were not included. The thermal dosage for each exposure class was calculated using the heat flux and the exposure time recorded in the sensors inside the protective clothing using equation 3 (Kinsman, 1991; Parsons et al., 2014), to assess the potential burn injury for each sensor over time:

(3) $$\mathrm{TDU}={\left(q_{i\,n}\right)}^{4/3}\times t$$

where *TDU* is Thermal Dosage Units [(kW⋅m^–2^)^4/3^⋅s], *q*_in_ is the incident heat flux (kW⋅m^–2^) and *t* is the exposure duration (s). Four thresholds of Thermal Dosage which have been identified to correlate with pain and burn injuries to exposed bare skin were used (O’Sullivan and Jagger, 2004): 92 (indicates onset of pain); 105 (represents onset of first degree burns), 290 (corresponds to second degree burns), and 1000 (corresponds to full depth third degree burns).

### Statistical Analysis

First a quality control of the heat flux data was performed, eliminating from the analysis the corrupted data revealing the failure of the data acquisition system, an open circuit pattern or directly the loss of the sensor. In the remaining records, the outliers were visually detected and replaced by the average value of the front and rear adjacent value. To reduce the noise still present in the signal, the Wavelet Shrinkage Denoising Method (Donoho and Johnstone, 1994) was executed for each of the four heat flux sensors’ signal. This method has shown to be more effective reducing noise than other traditional signal processing methods (e.g., Fourier transforms, moving average filter, Savitzky-Golay filter, etc.), since it preserves the original shape characteristics of the signal while improves the signal-to-noise ratio (Yang et al., 2009). Following the methodology proposed by Gradolewski and Redlarski (2014) denoising parameters were selected as: *Coiflets* wavelets family, with five decomposition levels, *minimax* threshold selection algorithm and *soft thresholding* with *mln* rescaling function. The denoising process was performed with the *wden* function of MATLAB R18b V.9.5.0 (MathWorks Inc., Natick, MA, United States).

The mean heat flux and temperature data were checked for normal distribution using the Shapiro–Wilk test. When normality was not fulfilled, a logarithmic transformation was performed. The mean and maximum heat flux, the exposure time and the exposure ratio were compared according to their position (i.e., chest vs. thigh) and their location (i.e., outer vs. inner) using a repeated two-way ANOVA with two within-subject factors (location × position). The assumption of sphericity was checked using the Mauchly’s test, if this assumption was violated the Greenhouse–Geisser adjustment was performed. When a significant *F*-value was found, Bonferroni’s test was used to establish significant differences between means. The comparison of the environmental temperature in the wildfire area vs. the temperature in the work environment was made using a Student’s paired *t*-test. The results are expressed as mean ± standard deviation (*SD*) except otherwise was stated. Values of *p* < 0.05 were considered statistically significant. SPSS V.22.0 statistical software (SPSS Inc., Chicago, IL, United States) was used.

## Results

**FIGURE 2:**
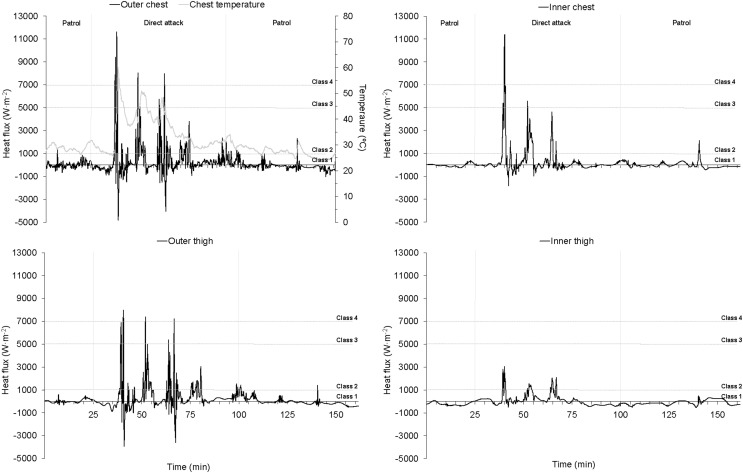
Heat flux and temperature profile during a representative intense wildfire. Class l: heat flux ≤ 1000 W⋅m^–2^; Class 2: >1000–≤5000 W⋅m^–2^; Class 3: >5000–≤7000 W⋅m^–2^; Class 4: >7000 W⋅m^–2^. The wildland firefighter wearing the data acquisition system performed patrolling and direct attack tasks. The heat flux and temperature data showed a great variability with fast oscillations and successive pulses of increase and decrease. The outer sensors showed a great variability in the heat flux values throughout the exposure, however, the exposure recorded in the inner sensors was less intense, obtaining a more stable pattern. The highest dose of thermal radiation was analyzed in this wildfire, obtaining a value of 110 (kW⋅m^–2^)^4/3^⋅s in the chest sensor.

Of the total wildfires recorded, only 23 were considered valid and were subsequently analyzed. Fifteen wildfires were discarded since the presence of misleading data and when failure in the signal was visually verified (i.e., connection with dataloggers was lost and/or the sensors were damaged or lost). The average suppression time was 186.9 ± 119.3 min. Overall, the heat flux at the inner sensors was 286.7 ± 255.0 W⋅m^–2^, with a maximum heat flux of 2370.4 ± 3004.5 W⋅m^–2^ (Table 1). The area weather temperature at fire location was significantly lower (*p* < 0.05) than the suppression environment temperature (24.6 ± 8.9°C vs. 32.6 ± 8.9°C), which reached a maximum value of 78.0 ± 8.9°C. The mean and maximum heat flux at the chest and thigh were significantly higher (*p* < 0.001) in the outer sensors compared to the inner ones (Table 1). This pattern, during a representative intense wildfire, is shown in Figure 2. The protective clothing attenuated the incident heat flux in a 69.9 ± 11.6%, showing a homogeneous pattern between the chest and the thigh. As expected, the exposure time was significantly higher (*p* < 0.05) in the sensors placed outside compared to those placed inside the protective suit (Table 1). In this regard, the exposure time in the outer sensor of the thigh was significantly longer (*p* < 0.05) than in the chest (Table 1).

**TABLE 1:** Average heat flux, maximum heat flux, exposure time, and attenuation factor of the protective suit during wildland fire suppression [mean ± SD (range)].

**Heat flux sensor**	**Heat flux (W⋅m^–2^)**	**Maximum heat flux (W⋅m^–2^)**	**Exposure time (min)**	**Exposure ratio (%)**	**Attenuation factor**	**Attenuation (%)**
Outer thigh	647.1 ± 451.8**^∗∗^** (70.4–1586.6)	5244.2 ± 3908.8**^∗∗^** (577.4–11614.0)	81.9 ± 71.0**^∗†^** (1.0–223.0)	58.7 ± 34.2**^∗†^** (1.1–100.0)	3.7 ± 1.1 (1.8–5.4)	71.6 ± 10.3 (43.8–86.9)
Inner thigh	236.9 ± 204.5 (29.1–729.9)	2111.5 ± 2631.4 (177.83–8477.8)	55.2 ± 61.4 (1.0–190.0)	29.1 ± 25.0 (1.1–82.6)		
Outer chest	655.8 ± 545.4**^∗∗^** (64.1–1892.5)	5361.6 ± 4571.3**^∗∗^** (175.5–12206.5)	64.3 ± 62.9**^∗^** (1.5–217.0)	33.9 ± 28.0**^∗^** (1.7–94.4)	4.4 ± 2.6 (1.8–9.2)	68.2 ± 17.1 (40.0–94.6)
Inner chest	316.5 ± 308.7 (27.1–989.0)	2995.8 ± 3555.7 (133.4–10971.0)	48.8 ± 57.5 (1.6–201.0)	24.5 ± 25.1 (1.4–87.4)		
Global average	464.1 ± 316.9 (53.8–1029.0)	3928.2 ± 3275.2 (598.8–9209.6)	62.5 ± 60.7 (1.4–206.4)	36.6 ± 21.0 (9.7–81.5)	4.0 ± 1.5 (2.6–6.9)	69.9 ± 11.6 (41.9–86.7)

*q, heat flux. **^∗^**Differences with inner sensor (p < 0.05). **^∗∗^**Differences with inner sensor (p < 0.001). **^†^**Differences with outer chest (p < 0.05).*

Table 2 shows the variables inside the protective suit, according to the thermal exposure classes. No differences were found in the heat flux nor the thermal radiation dose between the thigh and the chest. The values obtained in each exposure class were located in the lower part of the class interval. Only in class 1 the exposure time was significantly greater (*p* < 0.001) in the thigh compared to the chest. Considering the values of heat flux and exposure times in each class, the global weighted average of the heat flux was 497 ± 151 W⋅m^–2^ with a weighted average for thigh and chest of 388.8 ± 133.4 W⋅m^–2^ and 922.6 ± 180.4 W⋅m^–2^, respectively.

**TABLE 2:** Average values of heat flux, exposure time and thermal dose for inner sensors according to exposure thresholds.

		**Thigh**	**Chest**	**Average**
Class 1 (*q* ≤ 1000 W⋅m^–2^)	Heat flux (W⋅m^–2^)	168.9 l’ 101.9 (30.1–341.1)	186.6 l’ 120.6 (31.8–367.5)	178.4 ± 98.7
	Exposure time (min)	70.4 l’ 72.0 (0.9–255.2)**^∗^**	46.3 l’ 64.8 (1.0–217.1)	53.6 ± 59.9
	Thermal dose (kW⋅m^–2^)^4/3^⋅s	0.7 l’ 0.5 (0.1–1.7)	0.7 l’ 0.5 (0.1–1.5)	0.7 ± 0.5
	Number of fires	22	19	
Class 2 (1000 < *q* ≤ 5000 W⋅m^–2^)	Heat flux (W⋅m^–2^)	1524.2 l’ 340.7 (1017.8–2181.0)	1796.1 l’ 329.7 (1081.0–2124.0)	1634.0 ± 309.2
	Exposure time (min)	9.9 l’ 17.3 (0.1–59.0)	10.1 l’ 10.8 (0.1–32.1)	10.0 ± 14.5
	Thermal dose (k⋅m^–2^)^4/3^⋅s	10.8 l’ 4.9 (5.1–19.8)	11.4 l’ 2.8 (5.6, 14.4)	11.1 ± 2.6
	Number of fires	11	8	
Class 3 (5000 < *q* ≤ 7000 W⋅m^–2^)	Heat flux (W⋅m^–2^)	5857.9 l’ 551.1 (5387.6–6464.3)	6132.7 l’ 218.9 (5930.6–6365.2)	6045.4 ± 348.5
	Exposure time (min)	0.5 l’ 0.1 (0.1, 0.6)	1.6 l’ 1.8 (0.4–3.7)	1.0 ± 1.5
	Thermal dose (k⋅m^–2^)^4/3^⋅s	52.9 l’ 6.7 (47.2–60.2)	56.2 l’ 2.7 (53.8–59.1)	55.2 ± 4.2
	Number of fires	3	3	
Class 4 (*q* > 7000 W⋅m^–2^)	Heat flux (W⋅m^–2^)	7902.6	9381.1 l’ 786.1 (9056.0–11388.9)	9611.8 ± 913.5
	Exposure time (min)	0.2	2.0 l’ 2.2 (0.5–3.6)	1.4 ± 1.9
	Thermal dose (k⋅m^–2^)^4/3^⋅s	78.8	109.8 l’ 9.1 (95.3–110.2)	94.1 ± 14.7
	Number of fires	1	4	

*q, heat flux. **^∗^**Differences with chest (p < 0.05).*

## Discussion

These data provide the first time-resolved picture of wildland firefighter’s typical thermal exposure while suppressing real wildfires. Our results point out that at certain moments the heat flux can reach dangerous intensities capable to cause burn injuries (Figure 2). However, due to the duration of the wildfires and the variability of the exposure, the thermal load is lessened (heat flux, 464 ± 316.9 W⋅m^–2^; ambient temperature, 32.6 ± 8.9°C) and might be classified as light (Budd et al., 1997; Rossi, 2003; Willi et al., 2016). However, this environmental thermal load became a net heat gain that may significantly increase the wildland firefighters’ physiological heat strain (McLellan et al., 2013).

Previous studies (Budd et al., 1997) have described slightly lower temperatures (29°C) and higher heat fluxes (1600 W⋅m^–2^) than those obtained in this study. Our data show a wider range of exposure, both for the temperature (22 – 66°C vs. 19 – 35°C) or the heat flux (54 – 9200 vs. 700 – 8600 W⋅m^–2^) than the values reported by Budd et al. (1997). This suggests that environmental exposure in this study was more variable, possibly due to the methodological differences between the two studies. Budd et al. (1997) carried out prescribed burns in which the fuel load, the meteorological and the topographic conditions were homogeneous. On the contrary, our data were recorded in real scenarios, which meant a high heterogeneity in the suppression conditions. It has been previously reported the influence that topography, meteorological conditions, types, loads and humidity content of the fuels have in the heat emitted by flames (Zárate et al., 2008; Butler, 2014).

Other factors such as the measurement method and the type of work performed might have influenced the results. Previous studies (Budd et al., 1997) have used static sensors placed in the working area during the moments of most intense exposure. In contrast, in the present study sensors were placed on the personal protective equipment and the whole wildfire suppression event was recorded. Several studies (Eglin et al., 2004; Willi et al., 2016) have indicated that the thermal exposure reported by means of sensors held in fixed positions during fire-fighting training are not reliable since changes in the thermal environment in the area near the firefighter or in their protective clothing are not taken into account. Willi et al. (2016) analyzed the thermal environment of structural firefighters by means of sensors placed on the personal protective equipment during training fires and compared them with the values recorded by fixed sensors. These authors suggested that changes in the local temperature measured by fixed thermocouples does not provide a reliable indication of the changes in the firefighter’s local temperature. This might be related to the movement of firefighters toward less intense exposure areas, which cause a cooling (Willi et al., 2016). This work pattern has also been observed during real wildfire suppression (Rodríguez-Marroyo et al., 2011). Wildland firefighters regulate their exposure to heat by taking small breaks away from the fire in order to reduce the thermal load and the exercise intensity (Budd et al., 1997; Rodríguez-Marroyo et al., 2011).

Our results show that there is a certain asymmetry in how the heat flux in the chest and the thigh sensors is received (Table 1). Probably this is due to the type of tasks performed. In the analyzed wildfires direct attack was the main suppression tactic carried out, which means working with hand tools directly on the flames (between 0.5 and 1.5 m). In addition, once the fire was controlled, mop-up and blacking out tasks were conducted. In all these tasks the lower part of the body is exposed for a longer time to the incident heat flux, which might increase the exposure time of the thigh sensor.

The protective suit had an external heat flux AF of ∼70%. This result is similar to that found by Raj (2008) (50 – 70%) whom analyzed the protection factor of common wear (i.e., 100% cotton or 65% cotton and 35% polyester) in one or two layer configurations. Our results only show the AF offered by the protective suit, since the added protection of the underwear and the air gap between the clothing and the skin were not taken into account. It has been shown that thicker clothing, with a greater number of layers or with a looser fit (i.e., increased air gap) allows an increase in heat attenuation (Song et al., 2011). Despite this, the reduction in heat flux provided by the suit wore in this study (i.e., a single layer, ∼0.4 mm) was similar to that reported by Raj (2008) for configurations with two layers (i.e., ∼4 mm) or by Stoll and Chianta (1971) in an arrangement of a single layer apparel and a 4 mm air gap between the clothing and skin. This confirms that the synthetic fire-retardant fibers used to manufacture the wildland firefighters’ clothing offer a good protective behavior during real wildfires.

Results from Table 2 show that the thermal environment described in this study is clearly characterized by the lower exposure classes. Approximately 81% of the exposure time the heat flux was of ∼180 W⋅m^–2^ (Class 1), while 15% of the exposure time the incident flux was of ∼1600 W⋅m^–2^ (Class 2). Only 3.5% of the time heat flux was within high exposure classes, 3 and 4. Willi et al. (2016) analyzed the heat flux received in the helmet of structural firefighters during life-fire training exercises (∼11 min) in closed spaces. These authors found an average heat flux of 1600 W⋅m^–2^ (1000 – 2400 W⋅m^–2^). Severe thermal exposures were characterized by incident heat fluxes between 3000 and 6000 W⋅m^–2^, while moderate exposures had incident heat fluxes less than 1000 W⋅m^–2^. The time spent in each exposure class was of ∼3.5 min. These results show a more homogeneous and intense exposure than the recorded in our study. Only the maximum values (∼5000 W⋅m^–2^) obtained in the wildfires were in line with the severe thermal exposure range reported for structural firefighters (Rossi, 2003; Willi et al., 2016). In seven of the wildfires analyzed the absolute heat flux peaks achieved values above 10000 W⋅m^–2^, which meant a dangerous exposure (Rossi, 2003, 2016). However, these values were occasionally achieved meanwhile heat fluxes of this magnitude are attained more frequently in structural firefighters, reaching ∼30% of the exposure time (Willi et al., 2016).

To our knowledge, this work is the first which analyzes the vulnerability to thermal radiation during real wildfire suppression using the thermal dosage method. Thermal dose has been correlated to different burn injuries (Hymes et al., 1996). This variable integrates a measure based on magnitude and duration of heat exposure (Eisenberg et al., 1975) and has been used in different populations (Hockey and Rew, 1996; Daycock and Rew, 2000; O’Sullivan and Jagger, 2004). Recently, Parsons et al. (2014) conducted a series of laboratory scale numerical experiments examining potential burn injuries for wildland firefighters using the thermal dose method. This method is usually used to report the risk of second-degree burns (O’Sullivan and Jagger, 2004). Thermal exposure values capable to cause such type of burns were not reported in our study, since the highest average and maximum thermal dose values were ∼94 and ∼110 (kW⋅m^–2^)^4/3^⋅s, respectively (Figure 2). These values correspond to thermal dose thresholds of pain and first degree burns for infrared thermal radiation exposure (Hockey and Rew, 1996; O’Sullivan and Jagger, 2004). However, no burn injury was reported in this study. This fact might be related to the temperature of the skin since together with the intensity, duration and wavelength of heat source determine the response of humans to heat pain and injury (Wieczoreck and Dembsey, 2001).

The thermal exposure analyzed in this study supposed a net gain of heat, which added to the exercise effort performed during suppression (Rodríguez-Marroyo et al., 2012) may exacerbate the thermal and cardiovascular strain experienced by the wildland firefighters (Cuddy and Ruby, 2011). It might lead to a substantial increase in the sweating rate to compensate for the heat balance. Budd et al. (1997) reported an increase in the sweating rate from 793 g⋅h^–1^ to 1027 g⋅h^–1^ for an environmental heat load of 216 W (i.e., 115 W⋅m^–2^). Considering these results, one can speculate that the environmental heat load obtained in our study (∼460 W⋅m^–2^) might increase the sweat rate to ∼1400 g⋅h^–1^. This sweating rate might only be maintained for short periods (<1 h) in acclimated and correctly hydrated subjects (Cheuvront and Kenefick, 2014). This circumstance highlights the impact that the thermal work environment imposes on the physiological response of wildland firefighters.

## Conclusion

In conclusion, the mean thermal exposure analyzed in the present study may be considered light. However, high thermal exposure values may be obtained in punctual moments, which can elicit first-degree burns. Findings of this study highlighted the importance of protective clothing used by wildland firefighters. The behavior of this garment in real situations was very effective, since it attenuated the external heat flux by approximately 70%.

## Data Availability

The raw data supporting the conclusions of this manuscript will be made available by the authors, without undue reservation, to any qualified researcher.

## Author Contributions

BC-L and JL-S collected the data. BC-L and JR-M carried out the data analyses and wrote the manuscript. BC-L, JV, and JR-M interpreted the results. BC-L, JV, JL-S, and JR-M designed the study and approved the final version of the manuscript.

## Conflict of Interest Statement

The authors declare that this study received funding from Empresa de Transformación Agraria S.A. This funder is a public company, whose aims include the prevention and fight against wildfires. The company facilitated the access to the participants and contributed to acquire the protective suits and some sensors used in the study. The funder had no role in study design, data collection and analysis, decision to publish, or preparation of the manuscript. JL-S was employed by company TRAGSA. The remaining authors declare that the research was conducted in the absence of any commercial or financial relationships that could be construed as a potential conflict of interest.
